# Changes in Paraspinal Muscles and Facet Joints after Minimally Invasive Posterior Lumbar Interbody Fusion Using the Cortical Bone Trajectory Technique: A Prospective Study

**DOI:** 10.1155/2022/2690291

**Published:** 2022-01-12

**Authors:** Yue Li, Yuxiang Chen, Yuzeng Liu, Yong Hai, Xinuo Zhang, Li Guan, Tianqing Zhang

**Affiliations:** Departmen of Orthopedics, Capital Medical University Affiliated Beijing Chaoyang Hospital, Capital Medical University, No. 8 Gongtinanlu, Beijing 100020, China

## Abstract

In this prospective cohort study, we aimed to determine the surgical and adjacent segment changes in paraspinal muscles and facet joints in patients with lumbar spinal stenosis after minimally invasive posterior lumbar interbody fusion (PLIF) using the cortical bone trajectory (CBT) technique. We enrolled 30 consecutive patients who underwent the single-level CBT technique between October 2017 and October 2018. We evaluated preoperative and 1-month, 3-month, 6-month, and 1-year postoperative clinical data including Visual Analogue Scale (VAS) scores and Oswestry Disability Index (ODI). Magnetic resonance imaging (MRI) was performed a year after surgery. The erector spinae (ES) muscle area, volume, and fat infiltration (FI) on the surgical and adjacent segments were evaluated using the thresholding method, and the degree of adjacent facet joint degeneration was calculated using the Weishaupt scale. FI rate was graded using the Kjaer method. All patients underwent a 12-month follow-up. The VAS and ODI scores significantly improved after surgery in all patients. No patient showed degeneration of the adjacent facet joints (*P* > 0.05) during the 1-year follow-up postoperation. There was no significant difference in ES muscle volume, area, and FI on the surgical and adjacent segments (*P* > 0.05). The FI rate of the upper ES muscles increased postoperatively (*P* < 0.05); however, there were no significant changes in FI rate of the lower ES muscles. Patients with lumbar spinal stenosis could obtain satisfactory short-term clinical outcomes via minimally invasive PLIF using the CBT technique. Moreover, this technique may reduce the impact on the paravertebral muscles, especially the ES muscle, and the adjacent facet joints.

## 1. Introduction

Lumbar spinal stenosis (LSS) is a common spinal condition and the most frequent indication for spinal surgery in elderly people. Posterior lumbar interbody fusion (PLIF) surgery is a widely accepted surgical technique for the treatment of LSS [1]. Paravertebral muscles play an important role in maintaining lumbar spine stability [2]. Paraspinal muscle degeneration may lead to loss of functional muscle support, segmental movement disorders, and increased biomechanical strain, resulting in persistent postoperative low back pain [3]. Three main mechanisms have been proposed for structural changes in back muscles: disuse, denervation, and an active process mediated by a localized muscle inflammatory response [4]. Traditional pedicle screws point lateral to the pars interarticularis, and the operation lacks protection of the paravertebral muscles and requires a relatively wide dissection of the paraspinal muscles [5], which may predispose to injury to the medial and posterior branches of the spinal nerve and causes volume atrophy of the paravertebral muscles [1, 6]. Muscle degeneration is characterized by a decrease in muscle size and/or an increase in the amount of fat infiltration on magnetic resonance imaging (MRI) [7]. Moreover, the violation of the adjacent facet joint surface could lead to adjacent segment degeneration (ASD) [8]. Therefore, traditional PLIF with pedicle screws may cause paraspinal muscle injury and ASD, eventually leading to chronic low back pain.

In 2009, Santoni et al. introduced a new method for screw insertion called the cortical bone trajectory (CBT) technique [9]. This new trajectory follows caudocephalad and lateral paths in the sagittal and transverse planes, respectively, thereby increasing the purchase of the screw in the pedicle and vertebral body. Since the starting point of the screw is closer to the medial side, the incision and muscle separation lengths are also reduced [10]. Thus, shorter muscle dissection and incision lengths are associated with less parafacial muscle atrophy [11]. Moreover, a unique screw path reduces the violation of adjacent facet joints. In a previous study, the incidence of symptomatic ASD in the traditional PLIF group was approximately twice that in the CBT group [12]. Additionally, the incidence of symptomatic ASD was usually associated with paravertebral muscle injury and facet joint violation [13, 14]. However, few studies have assessed muscle injury and facet joint violation following the CBT technique based on MRI. Therefore, we aimed to investigate the changes in paraspinal muscles and facet joint degeneration after minimally invasive posterior interbody fusion using the CBT technique.

## 2. Materials and Methods

### 2.1. Study Design and Participants

We prospectively enrolled 30 consecutive patients with lesions at L4/5 from October 2017 to October 2018. LSS diagnosis was confirmed by MRI or computed tomography (CT). We included LSS patients (1) with severe low back and leg pains persisting after at least 3–6 months of conservative treatment and (2) without an obvious ASD on MRI before surgery. We excluded patients with (1) degenerative scoliosis (Cobb angle >10°), because scoliosis affects the calculation of muscle volume; (2) infection, trauma, or spondylolisthesis; and (3) severe psychosis who were uncooperative during follow-up.

This study complied with the Declaration of Helsinki and was approved by the Ethics Committee of Beijing Chaoyang Hospital, ID: 2017-KE-103. Participant informed consent was exempted because of the retrospective study design.

### 2.2. Surgical Technique

The patient was placed in a prone position, and an approximately 5 cm midline skin incision was made on lumbar. The muscles were separated layer-by-layer to expose the surgical site. Muscle was exposed to the exposed vertebral isthmus. The facet joints were exposed, avoiding the exposure of facet joints adjacent to the fused segment. Decompression was achieved by partial laminectomy and unilateral or bilateral facetectomy. The decompression-resected autogenous bone was made into bone blocks and filled into polyetheretherketone cages. After removing the disc and treating the superior and inferior endplates, the residual particulate bone was inserted into the anterior portion of the disc space, and the cage was subsequently inserted into the disc space. In the CBT technique, surgeons use screws typically measuring 5.5 mm in diameter and ranging from 35 to 40 mm in length. All procedures were performed by the same surgeon and there were no technical differences. All patients returned to normal activities after removal of the drainage tube. After discharge, low back muscle exercises were performed appropriately according to the rehabilitation.

### 2.3. Assessment Criteria

Pre- and postoperative parameters were assessed, including the degree of upper and lower facet joint ASD, the muscle area and volume, and the fatty infiltration (FI) rate of the adjacent and surgical segments of the erector spinae (ES) muscle.

FI rate was graded using the Kjaer method [7], “normal” for estimates of 0–10% fat within the muscle, “slight” for 10–50% fat, and “severe” for >50% fat. Upper and lower facet joints were assessed using the Weishaupt scale [15]. Grade 0 indicated normal facet joint space (2 ± 4 mm width). Grade 1 indicated narrowing of the facet joint space (<2 mm) and/or small osteophytes and/or mild hypertrophy of the articular process. Grade 2 indicated narrowing of the facet joint space and/or moderate osteophytes and/or moderate hypertrophy of the articular process and/or mild subarticular bone erosions. Grade 3 indicated narrowing of the facet joint space and/or large osteophytes and/or severe hypertrophy of the articular process and/or severe subarticular bone erosions and/or subchondral cysts. All parameters were measured on MRI. Facet joint ASD, at the same level, was expressed as the sum of the left and right facet joint Weishaupt scores.

**Figure 1:**
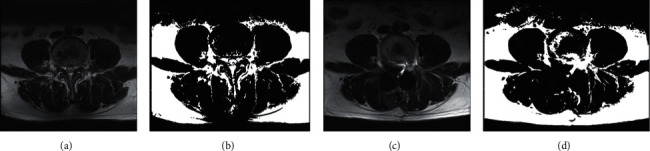
A 45-year-old male patient diagnosed with lumbar spinal stenosis. (a) represents L4-L5 vertebral MRI, while (b) is the same image obtained after processing by the ImageJ software. (c) represents L4-L5 vertebral MRI after CBT surgery, while (d) is the same image obtained after processing by the ImageJ software. The area enclosed by the yellow line after image thresholding in the ImageJ software is the ES portion of the paravertebral muscle. ES area and FI obtained by calculation using the ImageJ software.

**Figure 2:**
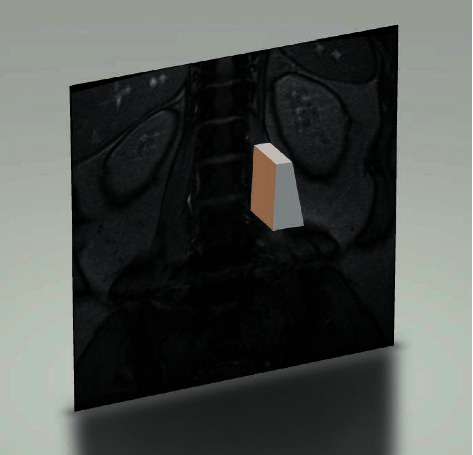
To estimate the volume, the entire ES muscle is considered as a circular table. The areas of the upper and lower segments are A and B, respectively. Using the height *h* between the upper and lower segments, the volume of the ES is estimated using the formula $V=\left(1/3\right)h\left(S_{A}+S_{B}+\sqrt{S_{A}S_{B}}\right)$.

ES muscle measurements were obtained from T2-weighted images using ImageJ software. The selection method for muscle regions of interest (ROI) was based on the technique proposed by Crawford et al. [16]. Based on fascial plane separation, the facet joint was used as a landmark between the multifidus and erector spinae muscles. A large fat-filled tent between the longissimus and iliocostal muscles was excluded from the ROI. In addition, fat areas lateral to the iliocostal and subfascial planes were excluded in the ROI. MRI was performed using Signa Hdxt 3.0t (Siemens). The slice thickness was 3 mm with a 3 mm gap between each slice, the parameters were set as FoV 200 mm, TR 2870 ms, and TE 87 ms. Patients were placed in the supine position, with their legs straight and the lumbar spine in a neutral position. Axial MRI was parallel to the inferior endplate of the vertebral body. Paraspinal muscle parameters were measured at the midpoint of the intervertebral disc. The surgical and adjacent segments were measured for each patient. Left and right values were summed, from which the average values for ES muscle area, volume, and FI were calculated. The ES muscle area and FI were measured using a thresholding technique (see Figure 1), while the ES muscle volume was estimated by multiplying the muscle area and height in the adjacent upper and lower regions (see Figure 2). The paraspinal muscles were regarded as approximate prisms, and the volume of the paraspinal muscles was calculated from a three-dimensional (3-D) perspective.

Clinical effects were assessed using the Visual Analogue Scale (VAS) score for back pain and the Oswestry Disability Index (ODI) evaluated preoperatively and at 1-month, 3-month, 6-month, and 1-year postsurgery. In VAS, pain is divided into 10 points, 2 points indicating no pain, 10 points indicating severe pain, and the middle part indicating varying degrees of pain. The patient was asked to place a mark on the horizontal line according to his/her feeling to indicate the degree of pain, 2–4 points for mild pain, 5–7 points for moderate pain, and 8-9 points for severe pain. The ODI covered 1 item on pain and 9 items on activities of daily living (personal care, lifting, walking, sitting, standing, sleeping, sex life, social life, and traveling). Each item was measured on a 6-point ordinal scale, ranging from the best scenario to the worst scenario.

### 2.4. Statistical Analysis

SPSS version 21.0 was used to analyze the collected data. All values were expressed as mean ± standard deviation. The Wilcoxon rank-sum test was used for grade data selection such as muscle FI and facet joint degeneration. Student's *t*-test was used to examine differences between groups of continuous variables such as muscle area, VAS, and ODI. *P* < 0.05 was considered statistically significant.

## 3. Results

Of the 30 patients enrolled, 16 were men while 14 were women, with an average age of 63.63 ± 9.51 years (range, 45–82 years). Single-level L4/5 PLIF was performed on all patients, respectively. The mean body mass index was 24.54 ± 3.83 kg/m^2^ and the average operation time was 153.33 ± 29.87 min. The mean intraoperative blood loss was 183.33 ± 69.89 ml, and the average hospital stay was 7.97 ± 2.20 days (see Table 1).

The mean preoperative and postoperative ODI scores and VAS scores are presented in Table 2, while the upper and lower segment ES muscle areas, surgical segment ES muscle areas, and ES muscle volumes are presented in Table 3. The VAS and ODI scores significantly improved after surgery in all patients. There was no significant difference between the preoperative and 1-year postoperative ES muscle area and volume (*P* > 0.05) (see Table 2). The median preoperative surgical segment ES muscle FI was 1 and the median 1-year postoperative surgical segment ES muscle FI was 1, the difference was not significant (*Z* = −1.41, *P*=0.16). The median preoperative upper segment ES muscle FI was 0.5 and the median 1-year postoperative upper segment ES muscle FI was 1, the difference was statistically significant (*Z* = −2.00, *P* < 0.05). The median preoperative lower segment ES muscle FI was 1 and the median 1-year postoperative lower segment ES muscle FI was 1, the difference was not significant (*Z* = −1.00, *P*=0.32).

**Figure 3:**
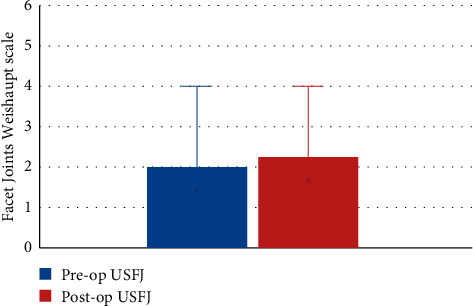
Preoperative and 1-year postoperative Weishaupt scale scores of the upper segment facet joints. USFJ, upper segment facet joints.

**Figure 4:**
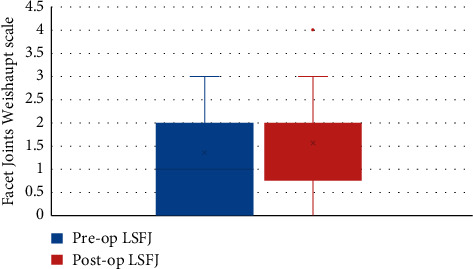
Preoperative and 1-year postoperative Weishaupt scale scores of the lower segment facet joints. LSFJ, lower segment facet joints.

There was no significant difference between the preoperative and 1-year postoperative facet joint scores. The median preoperative upper segment facet joint score was 1 and the median 1-year postoperative lower segment facet joint (LSFJ) was 2; however, the difference was not significant (*Z* = 2.45, *P* > 0.05) (see Figure 3). The median preoperative and 1-year postoperative LSFJ scores were both 2; however, the difference was not significant (*Z* = 1.89, *P* > 0.05) (see Figure 4).

## 4. Discussion

Our study mainly focused on the effect of the CBT technique on the ES muscle, a paravertebral muscle. The results showed that the CBT technique could adequately protect the ES muscle and facet joints during posterior open surgery. Moreover, it effectively relieved the patient's symptoms while protecting the ES muscle from destruction and avoiding the occurrence of persistent lower back pain caused by postoperative paraspinal muscle atrophy and ASD of the facet joints.

Paravertebral muscles play an important role in lumbar motion and maintenance of stability [17]. The paraspinal system mainly includes the multifidus and ES muscles. The ES muscle plays an important role in balancing the vertebral column. Previous studies have focused more on the multifidus muscle relative to the ES muscle. Öztürk et al. [18] found that in patients with low back pain caused by lumbar disc herniation, the ES muscle had more FI than the multifidus muscle. Paraspinal muscle atrophy indicates a reduction in the force generated by the ES muscles to support the basic load of the spine [19]. Some reports have indicated that the reduction in the cross-sectional area (CSA) of paravertebral muscles is associated with chronic low back pain [17, 20]. In the CBT technique, paravertebral muscle dissection is reduced because the entry point is closer to the midline. Therefore, the CBT technique is considered to have unique advantages in terms of reduced postoperative pain because of its smaller incision and less paravertebral muscle dissection. Hung et al. [21] found that patients who underwent CBT surgery had a smaller rate of the superior or inferior adjacent levels multifidus muscle FI than the pedicle screws group. Fan et al. [22] also demonstrated that the CBT techniques involved less paravertebral muscle dissection, less affected the paraspinal muscles, and better improved the postoperative VAS score than the traditional PLIF. This finding was like that of our study. The surgical and adjacent segment ES muscle areas did not change significantly in the 30 patients before and after surgery. Postoperative follow-up showed improvement in pain and quality of life (assessment by VAS and ODI). Thus, the CBT technique reduces interference with the ES muscle, which leads to the improvement of postoperative pain and function.

Previous studies on paraspinal muscles have mostly used two-dimensional (2D) analyses, by calculating the area of the paraspinal muscles. 2D analysis can only provide partial evaluation, whereas three-dimensional (3D) analysis, involving muscle volume calculation, more accurately evaluates the extent of muscle injury [23]. This study innovatively uses a 3D method of muscle volume estimation by measuring the cross-sectional area and the muscle length. This parameter better evaluates the effect of surgery on muscles. In the 30 patients, there was no statistically significant change in ES muscle volume after CBT surgery. No paraspinal muscle atrophy occurred during 1-year follow-up after surgery. This also demonstrates that CBT surgery has less effect on the paraspinal muscles.

In addition, unlike previous studies that assessed paraspinal muscle using CT, we used MRI to investigate the rate of paravertebral muscle FI. The results showed that the FI rate of the surgical and lower segments of the ES muscles did not change significantly after surgery. However, the rate of FI in the upper segment of the ES muscles increased at 1-year follow-up (*P* < 0.05). It has been shown that muscle swelling due to edema may persist for 10 months after surgery [21]. Therefore, to avoid the interference of edema, chronic FI changes should be evaluated more than 10 months after surgery [22]. Skin incisions for CBT surgery are usually small. Moreover, the contraction of the back muscles through this small skin incision increases the intramuscular pressure to a level that impedes local blood flow to the muscle and leads to muscle degeneration [24]. Small surgical incisions exert a greater tension when pulling surrounding tissue. Due to the learning curve relationship, the operation time and peri-incisional muscle traction time will be increased when the surgeon is inexperienced. Studies on the learning curve suggest that a shift in surgical technique and greater efficiency over time decreased the incidence of overall complications in the late cohort [25]. Both the operation time and the greater tension exerted by the small incision relative to pedicle screws can cause prolonged ischemia of the paravertebral muscles, which in turn causes postoperative muscle tissue lipidation. Surgeons in this study have completed more than 100 cases of CBT before performing this study and are proficient in surgical techniques with no impact on the study.

Facet joint violation is much lower in the CBT technique than in traditional techniques because the entry point of the former is near the midline, which is far from the superior facet joint [26, 27]. Facet joint violation has been reported to cause symptomatic adjacent segment disease and may affect the fusion rate after fusion surgery, resulting in low back pain [28]. Degenerative changes of the facet joints are often characterized by cartilage loss, subchondral bone sclerosis, and osteophyte formation. In this study, there was no statistically significant change in the 1-year postoperative facet joint scores of the 30 patients and no obvious facet joint degeneration was found during follow-up, demonstrating that the CBT technique effectively avoided interference with the upper and lower adjacent facet joints and avoided the occurrence of ASD. However, the follow-up time in this study was 1 year, and a longer follow-up may have different results.

Several limitations of our study should be acknowledged. First, the 1-year follow-up period may be too short to assess the long-term effect of the CBT procedure on pain relief and ASD. Second, our procedure focused only on single-level CBT surgery. The effects of long-segment CBT on the paraspinal muscles and adjacent segments need to be studied. Third, there was no comparison group for analysis. Comparison groups with conventional techniques should be included in subsequent studies for controlled studies to clarify the effects of CBT techniques on the paraspinal muscles. Fourth, postoperative low back muscle exercise is one of the effective measures to relieve postoperative paraspinal muscle fatty infiltration. The effect of surgical rehabilitation exercises on the paraspinal muscles was not focused on this study. Fifth, the learning curve of CBT techniques can also affect the paraspinal muscles. Surgical proficiency varies among surgeons at different stages of the learning curve. Surgeons in this study are already familiar with CBT techniques, but the effects of surgery on paraspinal muscles for different learning curve stages should be further investigated. Lastly, MRI generates incremental cost for the patient. This could cause patients to be lost to follow-up and increase the difficulty of long-term follow-up.

## 5. Conclusions

Our results showed that patients with lumbar spinal stenosis could obtain satisfactory clinical outcomes via minimally invasive PLIF using the CBT technique in the short run. Moreover, the aforementioned technique may reduce the impact on the paravertebral muscles, especially the ES muscle, and adjacent facet joints.

## Figures and Tables

**Table 1:** Characteristics of patients with lumbar disease in this series.

Characteristics	*n*
Sex	
Male	14 (46.7%)
Female	16 (53.3%)
Age	63.63 ± 9.51 years
Body mass index	24.54 ± 3.83 kg/m^2^
Operation time	153.33 ± 29.87 min
Intraoperative blood loss	183.33 ± 69.89 ml
Hospital stay	7.97 ± 2.20 days

**Table 2:** Clinical outcome assessment.

	Preoperative	1-month follow-up	3-month follow-up	6-month follow-up	1-year follow-up
ODI	77.63 ± 5.36	61.85 ± 8.65	50.52 ± 12.37	38.89 ± 10.56	34.70 ± 13.56
VAS	7.70 ± 0.65	5.03 ± 1.35	4.07 ± 1.09	3.23 ± 1.28	2.70 ± 1.21

VAS, Visual Analogue Scale; ODI, Oswestry Disability Index.

**Table 3:** Preoperative and postoperative paraspinal muscle parameters.

	Preoperative	1-year follow-up	*P*
USES area (mm^2^)	3168.14 ± 744.88	3215.08 ± 663.34	0.417
LSES area (mm^2^)	2989.21 ± 871.46	2968.72 ± 795.05	0.711
SSES area (mm^2^)	3495.66 ± 772.81	3463.48 ± 774.95	0.069
ESV (mm^3^)	192480.767 ± 45962.31	189865.65 ± 42912.18	0.384

USES, upper segment erector spinae muscle; LSES, lower segment erector spinae muscle; SSES, surgical segment erector spinae muscle; ESV, erector spinae muscle volume.

## Data Availability

The datasets generated and/or analyzed during the current study are not publicly available due to the data being confidential; however, they are available from the corresponding author on reasonable request.
